# Graphene on ferromagnetic surfaces and its functionalization with water and ammonia

**DOI:** 10.1186/1556-276X-6-214

**Published:** 2011-03-11

**Authors:** Stefan Böttcher, Martin Weser, Yuriy S Dedkov, Karsten Horn, Elena N Voloshina, Beate Paulus

**Affiliations:** 1 Fritz-Haber-Institut der Max-Planck-Gesellschaft, 14195 Berlin, Germany; 2 Institut für Chemie und Biochemie, Freie Universität Berlin, 14195 Berlin, Germany

## Abstract

In this article, an angle-resolved photoelectron spectroscopy (ARPES), X-ray absorption spectroscopy (XAS), and density-functional theory (DFT) investigations of water and ammonia adsorption on graphene/Ni(111) are presented. The results of adsorption on graphene/Ni(111) obtained in this study reveal the existence of interface states, originating from the strong hybridization of the graphene π and spin-polarized Ni 3*d *valence band states. ARPES and XAS data of the H_2_O (NH_3_)/graphene/Ni(111) system give an information regarding the kind of interaction between the adsorbed molecules and the graphene on Ni(111). The presented experimental data are compared with the results obtained in the framework of the DFT approach.

## Introduction

Graphene is a single layer of carbon atoms arranged in a honeycomb lattice with two crystallographically equivalent atoms (C1 and C2) in its primitive unit cell [1,2]. The *sp*^2 ^hybridization between one 2*s *orbital and two 2*p *orbitals leads to a trigonal planar structure with a formation of strong ∑ bonds between carbon atoms that are separated by 1.42 Å. These bands have a filled shell and, hence, form a deep valence band. The unaffected 2*p_z _*orbital, which is perpendicular to the planar structure of the graphene layer, can bind covalently with neighboring carbon atoms, leading to the formation of a π band. Since each 2*p_z _*orbital has one extra electron, the π band is half filled. The π and π* bands touch in a single point at the Fermi energy (*E*_F_) at the corner of the hexagonal graphene's Brillouin zone, and close to this so-called Dirac point, the bands display a linear dispersion and form perfect Dirac cones. Thus, undoped graphene is a semimetal ("zero-gap semiconductor"). The linear dispersion of the bands results in quasi-particles with zero mass, namely, the so-called Dirac fermions.

The unique "zero-gap" electronic structure of graphene, however, leads to a few limitations for application of this material in real electronic devices. In order, for example, to prepare a practical transistor, one has to have a graphene layer where energy band gap is induced via application of electric field or via modification of its electronic structure by means of functionalization. There are several ways of the modification of the electronic structure of graphene with the aim of gap formation [3]. Among these ways are (i) incorporation within the structure of nitrogen and/or boron or transition-metal atoms; (ii) use of different substrates that modify the electronic structure; (iii) intercalation of different materials underneath graphene grown on different substrates; and (iv) deposition of atoms or molecules on top, etc.

In this article, an attempt to modify the electronic structure of graphene via contact of this material with metal (ferromagnetic Ni substrate) and via adsorption of polar molecules (H_2_O, NH_3_) on top of the graphene/metal system is presented. These studies of water and ammonia adsorption on graphene/Ni(111) were performed via combination of experimental [angle-resolved photoelectron spectroscopy (ARPES), X-ray absorption spectroscopy (XAS)], and theoretical methods [density-functional theory (DFT) calculations]. XAS and ARPES studies of graphene/Ni(111) reveal the existence of the interface states, originating from the strong hybridization of the graphene π and Ni 3*d *valence band states with partial charge transfer of the spin-polarized electrons on the graphene π* unoccupied states. This leads to the appearance of induced magnetism in the carbon atoms of the graphene layer as confirmed by X-ray magnetic circular dischroism (XMCD). ARPES and XAS data of the H_2_O-NH_3_/graphene/Ni(111) systems enable us to discriminate between different strengths of interactions (physisorption or chemisorption), which appear between the adsorbed molecules and graphene on Ni(111). DFT calculations were used to model different geometries of the adsorbed molecules on top of graphene/Ni(111), and electronic structure calculations were performed for them. The results thus obtained and those of the previous theoretical studies are compared with the present experimental results.

The ARPES and XAS studies were performed on the BESSY UE56/2-PGM-1 and UE56/2-PGM-2 beam-lines, and MAX-lab D1011 beam-line, respectively. An ordered set of graphene overlayers was prepared on Ni(111) via thermal decomposition of propene (C_3_H_6_) according to the procedure described elsewhere [4-6]. The quality, homogeneity, and cleanliness of the graphene/Ni(111) system were verified by means of low-energy electron diffraction and core-level, as well as valence-band photoemission. Water and ammonia were deposited at the partial pressure of *p *= 5 × 10^-8 ^mbar on the surface of graphene/Ni(111) at 80 K, and the sample was kept at this temperature during spectroscopic measurements. XAS and XMCD spectra were collected at both Ni *L*_2,3 _and C *K *absorption edges in partial and total electron yield modes with an energy resolution of 80 meV. ARPES experiments were performed on experimental station allowing us to obtain 3D data sets of the photoemission intensity *I*(*E*_kin_,*k_x_*,*k_y_*), where *E*_kin _is the kinetic energy of the emitted photoelectrons, and *k_x_*, and *k_y _*are the two orthogonal components of the wavevector of electron. The energy/angular resolution in ARPES measurements was set to be at 80 meV/0.2°. The base pressure during all the measurements was less than 7 × 10^-11 ^mbar.

In our DFT studies, the electronic and structural properties of the graphene-substrate system have been obtained using generalized gradient approximation, namely the Perdew-Burke-Ernzerhof (PBE) functional, to the exchange correlation potential. For solving the resulting Kohn-Sham equation, we used the Vienna *Ab Initio *Simulation Package (VASP) with the projector-augmented wave basis sets [7]. The *k*-meshes for sampling the supercell Brillouin zone are chosen to be as dense as 24 × 24, when folded up to the simple graphene unit cell. Plane wave cutoff was set to a value of 875 eV.

**Figure 1:**
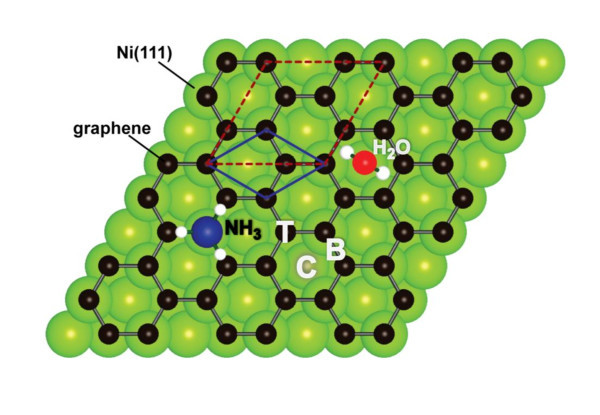
**Geometry of the H_2_O,NH_3_/graphene/Ni(111) systems investigated in this study**. Graphene layer is arranged in the *top-fcc *configuration on Ni(111). Adsorbed molecules can be placed in three different highly symmetric adsorption sites: T, *on-top*; B, *on-bond*; C, *center*, with respect to the graphene lattice. Two examples of adsorption are shown: for NH_3 _in the *on-top *position with hydrogen atoms directed to the neighboring carbon atoms, and for H_2_O in the *center *position with hydrogen atoms directed to the C-C bonds.

As was previously found [8] and confirmed in the present calculations, the most energetically advantageous arrangement is the *top-fcc *arrangement of carbon atoms on Ni(111) (see Figure 1). For this structure, several high symmetry adsorption positions for molecules are possible. They are T, *on-top*; B, *on-bond*; and C, *center *and are marked by the corresponding capital letters in Figure 1. There are up to 42 and 16 possible configurations of H_2_O and NH_3_, respectively, on top of graphene/Ni(111), but in our calculations, the authors restrict the choice to only six arrangements where molecules are placed in the high symmetry positions (T, B, and C) with hydrogen atoms pointing upwards (UP) or downwards (DOWN). Two examples of possible absorption geometries are shown for H_2_O (C-DOWN--hydrogen atoms are pointed toward the direction of C-C bond) and NH_3 _(T-UP--hydrogen atoms are pointed toward the direction of the neighboring C atoms) in Figure 1. In these experiments, molecular layers (MLs) of adsorbate with the thicknesses ranging from approximately one third to one fifth of the thickness of ML (corresponding to the dense packing of molecules, when one molecule is placed in every carbon ring) are studied. For simplicity, in the calculations of this study, the concentration of the adsorbed molecules was chosen as 1/3 of ML that corresponds to the (√3 × √3)*R*30° overstructure with respect to the unit cell of graphene (shown in Figure 1 as dashed- and solid-line rhombus, respectively).

**Figure 2:**
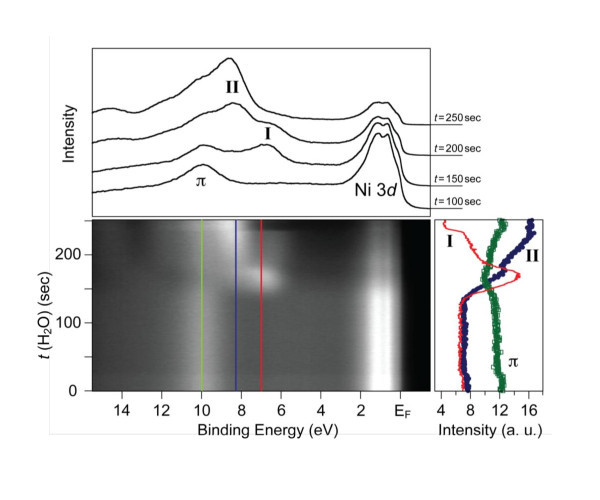
**(Central panel) Photoemission intensity map shows the modification of the valence band of the graphene/Ni(111) system at the Γ point upon adsorption of water molecules (partial water-pressure *p *= 5 × 10^-8 ^mbar; *t *is the deposition time)**. (Upper panel) Photoemission intensity profiles are shown for several time-points demonstrating the main photoemission features: Ni 3*d *states, graphene π states, and water-induced states (I and II). (Right panel) Photoemission intensity profiles as a function of water deposition time (*t*) taken at particular binding energies: red solid line, blue solid circles, and green open squares show intensity profiles at 7, 8.3, and 10 eV of the binding energies, respectively.

In order to study the growth modes of water or ammonia, the time sequences of the photoemission maps around the Γ point of the Brillouin zone (sampling angle of ±10° with respect to the normal emission) were recorded. The extracted photoemission intensity map showing the modification of the valence band at the Γ point of the graphene/Ni(111) system upon adsorption of water molecules (*t *is the deposition time) is shown in Figure 2 (central panel). Photoemission intensity profiles for several time-points demonstrating the main photoemission features of spectra [Ni 3*d *states, graphene π states, and water-induced states (I and II)], as well as intensity profiles as a function of water deposition time (*t*) taken at particular binding energies (red solid line, blue solid circles, and green open squares show intensity profiles at 7, 8.3, and 10 eV of the binding energies, correspondingly) are shown in the upper and right panels, respectively. The behavior of the water-related photoemission features, I and II, allows us to conclude that island-type growth of water on graphene/Ni(111) takes place: (i) These features start to grow simultaneously at *t *= 130 s, but slopes of the intensities growth are different; (ii) After *t *= 170 s, the intensity of feature I decreases via the exponential law, and there is a small plateau for the feature II (first ML is complete); (iii) At *t *= 230 s, when the thickness of deposited water is more than 2ML, probably, the structural phase transition takes place--formation of ice. Since ice is an insulator, the rapid decrease of the photoemission intensities of the Ni-related features and the shift of some states to higher binding energies can be explained by the formation of an insulating thin film of ice on top of the graphene/Ni(111) system. The delay in starting of the growth of the water-related photoemission features is somewhat puzzling (130 s until the first water-related signal appears in the spectra), but this delay could be because some clustering centers on the graphene/Ni(111) surface are necessary to allow water growth process to start. As soon as sufficient numbers of such centers are formed, the process of growth is accelerated. The general trend in the observation of the ammonia-related photoemission features in the similar experiments is the same. In subsequent XAS and ARPES experiments, the thicknesses of water and ammonia layers were chosen to be 1/3-1/2 of the ML (as discussed above with regard to the structure).

**Figure 3:**
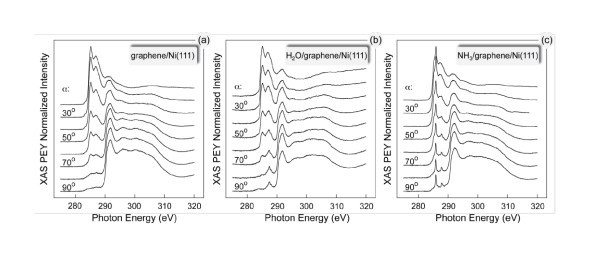
**XAS studies of water and ammonia adsorption on grapheme**. Angular dependence of the C *K*-edge XAS spectra of **(a) **graphene/Ni(111) and this system after adsorption of one-half of the ML of **(b) **H_2_O and (c) NH_3_, respectively.

The effects of the possible orbital mixing of the valence band states of the graphene layer on Ni(111) and orbitals of water and ammonia molecules were studied by XAS (Figure 3). This figure shows the angular dependence of the C *K*-edge XAS spectra of (a) graphene/Ni(111) and this system after adsorption of 1/2 of the ML of (b) H_2_O and (c) NH_3_, respectively.

The XAS spectra of the clean graphene/Ni(111) system (Figure 3a) were analyzed in detail in Refs. [6,9]. According to the theoretical calculations for this system, the first sharp feature in the XAS spectrum at 285.5 eV of photon energy is due to the transition of the electron from the C 1*s *core level to the interface state above the Fermi level (around the *K *point in the hexagonal Brillouin zone), which originates from the C *p_z _*- Ni 3*d *hybridization and corresponds to the antibonding orbital between a carbon atom C-*top *and an interface Ni atom. The second peak in the XAS spectrum at 287.1 eV of photon energy is due to the dipole transition of an electron from the C 1*s *core level to the interface state above the Fermi level (around the *M*-point in the hexagonal Brillouin zone) which originates from C *p_z _*- Ni *p_x_*,*p_y_*,3*d *hybridization and corresponds to a bonding orbital between C-*top *and C-*fcc *atoms, involving a Ni interface atom. As was found in the experiment, the observed hybridization leads to the orbital mixing of the valence band states of graphene and Ni and to the appearance of the effective magnetic moment of carbon atoms in the graphene layer. This moment was detected in the recent XMCD measurements of this system [6], which allow estimating the spin-magnetic moment of carbon in the range 0.05-0.1 μ_B _per atom.

The XAS spectra of the H_2_O/graphene/Ni(111) and the NH_3_/graphene/Ni(111) systems measured at the C *K *absorption threshold are shown in Figure 3b,c, respectively. These results demonstrate the controllable way of the graphene functionalization by water and ammonia. The corresponding adsorbate-induced states in the region of the unoccupied valence band states were detected (Figure 3: the photon energies in the region of 280-290 eV correspond to the C 1*s *→ π* transitions; the photon energies in the region of 290-320 eV correspond to the C 1*s *→ σ* transitions). In this context, it is worth emphasizing that the presented XAS measurements were recorded at the C *K *absorption edge and that they reflect (to some extent) the partial density of states of the carbon atoms in the system [10], and they clearly demonstrate the appearance of the orbital hybridization of the graphene-, water-, and ammonia-related states. The absence of the strong angular variations of the water- and ammonia-induced XAS signals might be explained by the statistically uniform distribution of the orientations of H_2_O and NH_3 _molecules on graphene/Ni(111).

The interpretation of the XAS spectra measured after water or ammonia adsorption can be performed on the basis of the peak-assignment, which has been presented above. For the water adsorbate, the new structure in the XAS spectra appears at the photon energy range corresponding to the hybrid state in the electronic structure of graphene/Ni(111) involving both carbon atoms in the unit cell of graphene and interface Ni atom. This leads to the assumption that water molecules are adsorbed either in the *center *or in the *on-bond *position on graphene/Ni(111) (Figure 1). Ammonia-induced spectral features in the C *K *XAS spectra are observed in the photon energy range corresponding to the hybrid state which is a result of hybridization of the *p_z _*orbital of the C-*top *atom and the 3*d_z2 _*state of the Ni interface atom. On the basis of this analysis, one can conclude that ammonia molecules are placed in the *on-top *position on graphene/Ni(111) with the lone-pair toward carbon atoms and N-H bonds along C-C bond of the graphene layer.

**Figure 4:**
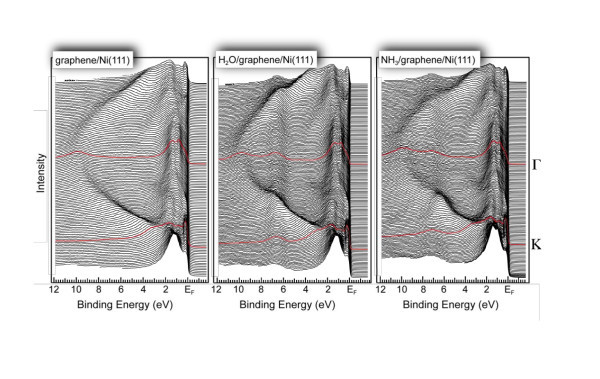
**Series of the ARPES spectra obtained on graphene/Ni(111), H_2_O/graphene/Ni(111), and NH_3_/graphene/Ni(111) along the Γ-*K *direction of the Brillouin zone**. The amounts of water and ammonia were estimated as 0.5 of the ML. These data were collected with the photon energy of 75 eV.

Figure 4 shows a series of ARPES collected with the photon energy *hν *= 75 eV along the Γ-*K *direction of the Brillouin zone for the graphene/Ni(111), H_2_O/graphene/Ni(111), and NH_3_/graphene/Ni(111) systems. In all the series, one can clearly discriminate the dispersions of graphene π- and σ-derived states in the region below 2 eV of the binding energy as well as Ni 3*d*-derived states near *E*_F_. The binding energy difference of ≈2.4 eV for the π states and ≈1 eV for the σ states in the center of the Brillouin zone (in the Γ point) between graphite and graphene on Ni(111) is in good agreement with previously reported experimental and theoretical values [4,5,8], and it is explained by the differential strengths of hybridization for π and σ states in relation with Ni 3*d *states. The effect of hybridization between Ni 3*d *and graphene π states can be clearly demonstrated in the region around the *K *point of the Brillouin zone: (i) one of the Ni 3*d *bands at 1.50 eV changes its binding energy by ≈150 meV to larger binding energies when approaching the *K *point; (ii) a hybridization shoulder is visible in photoemission spectra which disperses from approximately 1.6 eV to the binding energy of the graphene π states at the *K *point. The full analysis of the electronic band structure and magnetic properties of the graphene/Ni(111) system were performed in Ref. [9].

The adsorption of 1/2 of ML of water and ammonia molecules on graphene/Ni(111) leads to the appearance of the additional photoemission signal in the spectra at 6.5 and 7.3 eV, respectively (Figure 4). In these spectra, these emissions are associated with the H_2_O-3*a*_1 _and NH_3_-1*e *states, respectively. As can be clearly seen from the photoemission spectra, the adsorption of H_2_O or NH_3 _on graphene/Ni(111) leaves the electronic structure of graphene π- and Ni 3*d*-states almost intact. This observation can be taken as an indication of the inertness of the graphene layer on Ni(111) as was earlier demonstrated in Ref. [4]. There are only small changes of the electronic structure of graphene/Ni(111) upon adsorption of water or ammonia. The small shift of about 150 meV of the graphene π band to the small binding energies is detected at the Γ point of the Brillouin zone in both cases. At the *K *point, there is a shift of this band to the higher binding energies of about 50 and 70 meV for the water and ammonia adsorptions, respectively.

Thus, ARPES and XAS data allow us to refer the interaction between considered molecules and the graphene/Ni(111) system as physisorption. From a theoretical point of view, physisorption can be considered as weak interaction arising due to two types of forces, namely, dispersion forces and/or classical electrostatic ones. The dispersion interactions are long-range electron correlation effects, which are not captured in DFT because of the local character of common functionals. Consequently, DFT often fails to describe physisorption correctly. For a correct and consistent treatment of physisorption interaction, it is necessary to use high-level wave-function-based post-Hartree-Fock methods like the Møller-Plesset perturbation theory [11] or the coupled-cluster (CC) method [12]. One problem here is that a very accurate treatment, e.g., with the CC method, scales very unfavorably with the number of electrons in the system. In general, this difficulty is avoidable by employing the so-called method of increments, where the correlation energy is written in terms of contributions from localized orbital groups [13]. An alternative approach is an inclusion of the dispersion correction to the total energy obtained with standard DFT approximation explicitly by hand with, e.g., DFT-D method, that is atom pair-wise sum over C_6_R^-6 ^potentials (see, e.g., Ref. [14]).

Recently, studies based on first principles for single H_2_O molecule adsorbed on freestanding graphene were performed by O. Leenaerts *et al. *[15]. For comparison purpose, the reported interaction energies (*E*_int_) are listed in Table 1 together with the corresponding equilibrium distances (*d*_0_). (The VASP-calculations of this study for (3 × 3) supercell yield similar values). One can observe very low interaction energies and no energetic preference regarding the adsorption site or orientation of the adsorbate. We have repeated the calculations taking into account the dispersion correction as proposed by Grimme [16] (DFT-D2 method). The resulting interaction energies are higher by 4-7 times in magnitude, although still physisorption is predicted coincidently with experimental observations. Consequently, the equilibrium distances between H_2_O and graphene are significantly shorter. In addition, DOWN orientation is clearly more preferred in this case as compared to the opposite one (i.e., UP). It can be noted that the obtained results are in reasonable agreement with the recent CCSD(T) data evaluated for the H_2_O/graphene system. Thus, for further consideration of the systems of interest, the PBE-D2 approximation will be used.

**Table 1:** The interaction energies (*E*_int_) and the equilibrium distances (*d*_0_) between H_2_O and the surface of the freestanding graphene layer as obtained for the six selected geometries at DFT level with standard PBE functional and when including dispersion correction (PBE-D2)

Geometry	PBE^a^		PBE-D2	
	*d*_0 _(Å)	*E*_int _(meV)	*d*_0 _(Å)	*E*_int _(meV)
C_DOWN	4.02	19	2.60	139
C_UP	3.69	20	3.07	83
B_DOWN	4.05	18	2.67	129
B_UP	3.70	18	3.17	77
T_DOWN	4.05	19	2.64	127
T_UP	3.70	19	3.18	75

^a^Data taken from Ref. [15].

The results obtained for the (√3 × √3)*R*30° overstructures of adsorbed molecules on graphene/Ni(111) are presented in Table 2. Owing to the symmetry breaking by the Ni(111) support, two inequivalent carbon atoms in *on-top *positions have to be considered in these cases. The difference between adsorption behaviors of water on graphene and graphene/Ni(111) indicates the effect of the substrate underneath of the graphene layer, and can be explained by the fact, that in the latter case, the electron charge density is shifted to the interface between the graphene layer and the Ni(111) support. At the same time, similar to the case when free-standing graphene is used as a substrate, for the H_2_O/graphene/Ni(111) system, DOWN orientation is the energetically most favorable one and the preferable adsorption site is the *center *of the carbon ring. This theoretical observation confirms our prediction based on the interpretation of XAS spectra. It can be noted that during these calculations, structural optimization of the system was not preformed, and only the distance between graphene and adsorbate is relaxed. Full optimization of H_2_O geometry in the case of C_DOWN configuration leads to *d*_0 _= 2.51 Å, that is a deviation of 2% with respect to the non-relaxed value. The corresponding interaction energy is lower by 4%, than *E*_int _given in Table 2.

**Table 2:** The interaction energies (*E*_int_) and the equilibrium distances (*d*_0_) between H_2_O (NH_3_) and the graphene/Ni(111) substrate as obtained for the eight selected geometries at PBE-D2 level of theory

Geometry	System			
	
	H_2_O/graphene/Ni(111)		NH_3_/graphene/Ni(111)	
	
	*d*_0 _(Å)	*E*_int _(meV)	*d*_0 _(Å)	*E*_int _(meV)
C_DOWN	2.55	123	3.19	127
C_UP	3.03	64	2.93	143
B_DOWN	2.64	111	3.21	124
B_UP	3.11	58	2.95	141
T(C1)_DOWN	2.63	110	3.12	123
T(C1)_UP	3.14	56	2.89	148
T(C2)_DOWN	2.62	111	3.12	125
T(C2)_UP	3.13	58	2.91	146

For the most stable arrangement of H_2_O on top of graphene/Ni(111), the band structure calculations were performed. One finds the H_2_O-related states at the following binding energies: 3.97, 5.96, and 9.85 eV, which satisfactorily match the APRPES data.

One can see, when looking at data listed in Table 2, that in the case of ammonia, its interaction energy with the substrate is higher compared to the values obtained for the H_2_O/graphene/Ni(111) system, which is also in good agreement with the experimental results, where the modification of the XAS C *K *spectra was observed. In this context, the UP orientation is preferable for any adsorption position. Although *on-top *(T_C1) adsorption yields the highest interaction energy, one has to be aware that the present calculations cannot give exact answer regarding the energetically most favorable adsorption position since the obtained interaction energies are very close to each other (within 3%). Geometry optimization can make this difference more pronounced, especially when taking into account stronger interaction between ammonia and the considered substrate.

Overall, from a theoretical side, one can see good agreement between the experimental data and the ones obtained by means of DFT calculations. However, further investigations are required before making the final conclusion regarding the position and orientation of the adsorbate with respect to the substrate under study. First, all possible arrangements of H_2_O and NH_3 _on top of graphene/Ni(111) have to be considered. Optimization of molecular geometry as well as relaxation of interlayer distances within the substrate has to be performed. Furthermore, parameter-free way of accounting for dispersion corrections is preferable. The latter is possible via van der Waals density functional, developed by Dion et al. [17].

In conclusion, the authors have studied the modification of the electronic structure of the graphene/Ni(111) system upon adsorption of water and ammonia molecules at low temperature. Adsorption of both types of adsorbates leads to the modifications of the XAS C *K*-edge spectra indicating the orbital mixing of the valence band states of graphene and adsorbates. For the occupied states, the small shifts of the graphene π states were detected in both cases with overall shift of the graphene π states to the lower binding energies reflecting the effect of *p*-doping (with respect to the initial state) after adsorption of water and ammonia on graphene/Ni(111). Analysis of experimental results leads us to the idea of the site-selective adsorption: water is adsorbed either in the center of carbon ring or on the bond between two carbon atoms; ammonia molecules are adsorbed on the carbon atom, which is located above the Ni interface atom. This assumption is supported by the results obtained via DFT calculations.

## Abbreviations

ARPES: angle-resolved photoelectron spectroscopy; DFT: density-functional theory; PBE: Perdew-Burke-Ernzerhof; VASP: Vienna *Ab Initio *Simulation Package; XAS: X-ray absorption spectroscopy; XMCD: X-ray magnetic circular dischroism.

## Competing interests

The authors declare that they have no competing interests.

## Authors' contributions

SB, MW and YSD carried out the experiment and perform treatment of experimental data. SB and ENV performed the calculations. YSD conceived of the study, and participated in its design and coordination. KH participated in design and coordination of the experimental part of this study. BP coordinated the theoretical part of this study. YSD and ENV prepared the manuscript initially. All authors read and approved the final manuscript.
